# Understanding Students’ Instrumental Goals, Motivation Deficits and Achievement: Through the Lens of a Latent Profile Analysis

**DOI:** 10.5334/pb.265

**Published:** 2016-07-13

**Authors:** Luke K. Fryer, Anja Van den Broeck, Paul Ginns, Kaori Nakao

**Affiliations:** 1 Hong Kong University, Centennial Campus CPD 180, Hong Kong; 2 Faculty of Economics and Business, KU Leuven, Belgium and Optentia, North-West University, South Africa; 3 Faculty of Education and Social work, University of Sydney, Australia; 4 Language Education Research Centre, Kyushu Sangyo University, Japan

**Keywords:** Instrumental goals, Multiple goals, Future orientation, Goal regulation

## Abstract

Building on the future oriented and regulated nature of instrumental goals, Lens and colleagues developed a 2 (proximal-distal) x 2 (internal-external) motivational framework. The current study aimed to test this framework from a person-centred perspective, while equally taking into account students’ lack of motivation as to extend the empirical and theoretical borders of the model. Latent Profile Analyses were used to test the viability of two to five motivational profiles among Japanese second-year students (*N* = 781). A solution with three latent subgroups fitted the sample best, explaining 6% to 62% of the variance in the measured variables. The profiles were labelled “low future oriented motivational profile”, “average motivated profile”, and “highly motivated profile”. The highly motivated subgroup reported the most adaptive pattern of motivation and highest levels of deep level learning, while few differences were found for surface learning and GPA. Theoretical and practical implications are discussed.

Why do some students learn and perform well, while others do not? Many factors are essential to student success, but arguably one of the most important factors is students’ motivation. Focusing the lens on motivation, there is a growing understanding that an array of simultaneously experienced motivations play a role in student learning (e.g., Pintrich, 2000; Barron & Harackiewicz, 2001).

Students might have a number of relevant answers for (not) persisting and therefore achieving or failing to achieve (Hidi & Harackiewicz, 2000). Students’ reasons might be more external to their studies and range from proximal goals such as course credits and graduation, to distal goals like financial and career-related life goals (Simons, Vansteenkiste, Lens, & Lacante, 2004). Students might also cite more personal or internal reasons, including proximal reasons such as (lack of) effort, interests, or peer relationships, or more distal reasons such as future personal development and quality of life after university (Lens, 2001). Educational research has indicated that a range of factors can explain students’ study efforts and achievement. It remains obscure, however, how various reasons are experienced in tandem and how they relate to academic success.

The current study aims to address this gap and focuses on students’ pursuit of multiple instrumental goals (i.e., Lens, Simons, & Vansteenkiste, 2004; Simons, Dewitte, & Lens, 2003, 2004; Simons, Dewitte, & Lens, 2000) for learning during university, thereby also including a measure of students’ concurrent amotivation related experiences. Specifically, we aim to examine how the pursuit of these goals and amotivation are experienced together, and what the correlates of these motivational profiles might be in terms of the depth of student learning and the essential outcome of achievement. Furthermore, the current study builds on previous research by focusing on the under-studied context of learning at a Japanese university. The current research therefore had an opportunity to expand the cultural borders of the research domain Lens and colleagues inspired.

## Instrumental motivation

Lens and colleagues have modelled instrumental motivation in terms of future orientation and quality of regulation (Simons, Dewitte, & Lens, 2004; Lens, 2001; Simons, Dewitte, & Lens, 2000). Essential to understanding the role of an individual’s orientation towards the future is Future Time Perspective (FTP). FTP refers to “a) the degree to which and b) the way in which the chronological future (proximal versus distal) is integrated into the present life-space of an individual through motivational goal-setting processes” (Husman & Lens, 1999, p. 114). FTP is an essential element of instrumental goals, as such goals are by definition future oriented. For example, if students study for the sake of studying in itself, they are said to have a task goal. If they study to achieve outcomes outside of the activity such as a diploma or gain the necessary competencies to become a good professional, they are said to focus on the utility of the task and to have instrumental goals.

With regard to quality of regulation, several scholars have differentiated qualitatively different types of motivation, the oldest one being the differentiation between intrinsic and extrinsic motivation (e.g., Deci, 1971). Today, this dichotomy of intrinsic versus extrinsic motivation has been superseded by theory modelling different types of extrinsic motivation within Self-Determination Theory (SDT; Deci & Ryan, 1985). Specifically SDT differentiates extrinsic motivation in terms of externally regulated (i.e., engaging in a behaviour because you feel controlled by others), introjected regulation (i.e., engaging in a behaviour because you pressure yourself) and identified regulation (i.e., engaging in a behaviour because you think it is important). While the former two types of motivation are considered to be controlling because they are external to the self, identified regulation is considered to be autonomous and more volitional in nature, just like intrinsic motivation. Seen from Deci and Ryan’s model, whether future oriented motivation is adaptive, depends on its regulation: Whether an individual perceives the future goal as being autonomously or controlled regulated.

Working to reconcile the pervasive and potentially adaptive nature of the instrumental goals, fuelled by FTP and modern perspectives on motivation of SDT, Lens and colleagues first developed a model which differentiated instrumental goals according to their perceived utility (near/distant) and regulation (internal/external) (Lens, 2001; Simons, Dewitte, & Lens, 2000). Working within this 2 × 2 framework, high utility and self-determined goals were shown to be the most adaptive ones as they were positively related to adaptive learning behaviours and key variables such as intrinsic motivation and interest. Extending this research, Simons, Dewitte and Lens (2004) modelled the temporal distance and regulation of students’ instrumental goals, employing a similar 2 (proximal/distal) × 2 (internally/externally regulated) framework. Results showed that instrumental goals that were internally regulated and distal were substantial predictors of adaptive motivations and behaviours such as excitement, persistence, good study habits and deep processing of materials. These positive effects could be attributed to the fact that internal distal goals stimulated students to adopt mastery goals (Simons, Dewitte, & Lens, 2004).

The 2 (internally/externally regulated) X 2 (distal/proximal) framework discerns four hypothetical goal types as distinct measurable latent constructs (Fryer, 2013). Pilot studies in both departmental learning (i.e. learning across students’ departmental courses; Fryer, 2013) and specifically learning English as a foreign language course (Fryer, Carter, Ozono, Nakao, & Anderson, 2013), however, only resulted in three of the four hypothesised constructs. The three instrumental goal types were 1) proximal externally regulated goals, which are temporally proximal goals, 2) distal externally regulated goals, and 3) distal internally regulated goals.

In particular, proximal externally regulated goals referred to students’ curricular aims for the current semester or year; these were goals which were less likely to have a strong internal locus of causality (e.g. passing a course). Second, distal externally regulated goals were temporally further in the future (e.g. after university is finished) and are generally externally controlled (e.g. getting a high payed job). Finally, distal internally regulated goals were also temporally distant, mostly referring to time after university but have strong internal regulation, such as self-development and contributions to society.

Notably, the proximal internal goals from Lens’ 2 × 2 framework did not have sufficient convergent and divergent validity to be used in causal modelling. However, in addition to the three goals validated from the original 2 × 2 framework, a fourth goal type also arose. For this goal type all social-related items (teacher, peer and parent) appeared to form a distinct, independent factor. The items defining the new socially instrumental goal factor were originally constructed to contribute to either proximal or distal externally regulated goals, but now seemed to lead to a separate factor. This socially instrumental goal construct was later replicated with a second sample (Fryer, 2013) suggesting that it was not spurious. While the first three goal types were consistent with Lens and colleagues original framework, social goals appeared to be specific to the context/sample. These findings were in alignment with past research within achievement goal theory (i.e., Urdan & Maehr, 1995)

Longitudinal modelling was a necessary step to test and extend the theoretical and cross-sectional research undertaken by Lens and colleagues. Subsequent cross-lagged modelling with the four instrumental goals measured by Fryer (2013)—distal internally regulated, distal externally regulated, proximal externally regulated goals and socially instrumental goals—broadly supported many of Lens et al.’s hypotheses and cross-sectional findings. In subsequent research within students’ general departmental learning (Fryer, Ginns, Walker, 2014), distal internally regulated goals were substantial predictors of future learning behaviours (deep approaches to learning), both directly and mediated by task goals. The other two goal types were broadly maladaptive in their contribution to future learning. Subsequently, Fryer (2015) found, consistent with Lens (2001), that distal internally regulated goals were strong predictors of future interest. Distal externally regulated goals, however, were a non-significant (*p* > .05) predictor and proximal externally regulated goals had a statistically significant small negative contribution.

The variable-centred studies presented to this point, have pointed to the positive role of future-oriented goals, and particularly those related to the students themselves, as opposed to externally regulated goals. This is also in line with Self-Determination Theory, providing empirical support for the importance of autonomous compared to controlled motivation (Ryan & Deci, 2000).

Although highlighting the relative importance of the differentiated goal types, variable-centred studies do not take into account that people may simultaneously pursue different goals and, hence, cannot speak to the diverse pursuit of multiple goals that subgroups have within a population (i.e. Pintrich, 2000). To add evidence to this area in the literature, this study relies on *motivational profiles* to examine students’ simultaneous experience of multiple motivations along with key learning outcomes and GPA.

## Profiling student motivation

Breaking rank with longstanding theory suggesting that one type of goal is better than another, Pintrich (2000) indicated that multiple goal pursuit is important for student learning and achievement. His research has been followed by a range of other studies (e.g., Wentzel, Baker, & Russell, 2012; Boekaerts, de Koning, & Vedder, 2006; Barron & Harackiewicz, 2001; Suárez Riveiro, Cabanach, & Arias, 2001) and has resulted in a broad acceptance that individuals can simultaneously pursue a number of goals. Furthermore, it has been theorised that some goals might work jointly towards adaptive outcomes. For example, Pintrich (2000) suggested that task and performance goals might work together towards enhanced achievement. One means of examining the convergence of students’ simultaneous pursuit of different (instrumental) goals are person-centered profile-analyses.

Profiling may shed light on how different types of motivations are simultaneously experienced and their combined relationship with learning processes and achievement. Profiling has supported our understanding of the different effects of the quantity and quality of motivation (e.g., Vansteenkiste, Sierens, Soenens, Luyckx, & Lens, 2009). It might therefore also prove to extend variable centered work examining the future oriented dimension of motivation.

For example, based on Self-Determination Theory’s conception of motivational quality, Vansteenkiste, and colleagues (2009) used cluster analysis to contrast the competing assumptions about the quantity and quality of motivation in the context of high school and university. Based on the type of motivational regulation, they hypothesized, and found, four fundamental profiles: one high in only autonomous motivation (high quality), one high in only controlled motivation (low quality), one high in both autonomous and controlled motivation (high quantity) and one low on both types of motivation (low quantity). In line with SDT, these authors found the high quality (autonomous) profiles to be broadly adaptive, with the clearest linkages to important learning outcomes (e.g., GPA, effort, and learning strategies). Other profiling research in the area of motivation using autonomous motivation found evidence for only three profiles, characterised by low, average and high levels of motivation (Ratelle, Guay, Vallerand, Larose, and Senécal, 2007). Similarly, using intrinsic and extrinsic motivation Corpus and colleagues provided evidence for both a 4-cluster solution (2012) and a 3-cluster (2014) solution among high school and elementary school children (respectively). The present study aimed to add to and expand on the current profiling research, thereby relying on Lens and colleagues’ 2 (distal/proximal) x 2 (internally-/externally-regulated) framework and by addressing Vansteenkiste et al.’s (2009) call for the inclusion of amotivation in such profiling analyses. Vansteenkiste et al. suggested that low quantity motivation groups might emerge as a mixture of amotivation and low autonomous/controlled motivation. It might be that by including a measure of students’ deficits in motivation, explicitly allowing for the emergence of a group of students’ with no motivation at all (see Haerens, Kirk, Cardon, De Bourdeaudhuij, & Vansteenkiste. 2010), we might improve our understanding of students’ instrumental goal pursuits.

## Amotivation and its dimensions

A considerable amount of research has examined the nature and role of amotivation. Quantitative research on amotivation began with the work of Vallerand and colleagues (1992) and follow up research showed that amotivation was important to predict outcomes such as (the lack of) environmental action (Pelletier, Dion, Tucson, & Green-Demers, 1999) and high school dropout (Vallerand, 1996). In line with its importance, Ratelle et al.’s (2007) included amotivation as a general construct in their profile analysis. With multiple samples, Ratelle et al. observed a consistent pattern of three sub-groups. Two groups exhibited high or moderate amounts of autonomous motivation paired with consistent levels of controlled motivation and very low levels of amotivation. The third group had lower levels of autonomous and controlled motivation with the highest levels of amotivation.

Seeking to gain a more refined insight into the notion of amotivation, Legualt, Green-Demers and Pelletier (2006) developed and employed a multi-dimensional model of amotivation. Building on previous research and theorizing (e.g. Skinner, Wellborn, & Connell, 1990; Bandura, 1977), they included four types of motivational deficit: 1) task characteristics which describe students’ lack of motivation due the nature of learning tasks; 2) task valuation which describe students’ lack of value for learning; 3) ability beliefs which describe their perceived lack of ability to successfully undertake the learning; 4) effort beliefs which describe why students fail to study due to a lack of energy or willingness to persist. Together these four constructs were modelled as amotivation. Green-Demers and colleagues (2008) later validated the multi-dimensional model. Adaptation to the context of Japanese higher education (Fryer, 2013), however, resulted in just three of these dimensions having sufficient convergent and divergent validity within simultaneous modeling: value, ability and effort beliefs. Past variable- (Fryer, 2013) and person-centred (Fryer, Bovee, & Nakao, 2014) analyses have provided evidence for the convergent validity of these three constructs.

## The Current Study

Past person- and variable-centred studies have examined a wide range of covariates for subgroups based on motivational regulation. For example, subgroups experiencing differing types of motivation have been found to differ in study strategies (Vansteenkiste et al., 2009) and academic achievement (Corpus & Wormington, 2014). The current study seeks to examine these learning outcomes using profiles based on distal versus proximal and externally versus internally regulated goals as well as amotivation. Past variable-centred research has suggested that self-reported deep and surface learning strategies are important learning outcomes related to students’ instrumental (i.e., externally-regulated) goals (Fryer et al. 2014, Simons et al. 2004). Adding GPA provides thus a source of external validity to the results, as previous person-centred research has found that autonomous, high quality goal profiles are associated with higher achievement (e.g., Vansteenkiste et al., 2009; Ratelle et al., 2007).

In addition to understanding key educational outcomes to the profiled variables, the role of gender needed to be explored. Longstanding reviews of the field (e.g., Meece, Glienke, & Burg, 2006), and a recent meta-analysis (Voyer & Voyer, 2014), has highlighted that female students often experience more adaptive motivation and obtain higher grades. Person-centered studies have supported these findings (Vansteenkiste et al. 2009; Ratelle et al., 2007): Female students were over-represented in adaptive sub-groups. We aimed to compare these findings with our profiling results.

The current study aimed to add to the emerging literature in the area of instrumental goal research by combining Lens and colleagues’ 2 × 2 framework with students’ amotivation for their studies. Based on past person-centred research in this general area (e.g., Corpus & Wormington, 2014; Wormington & Corpus, 2012; Vansteenkiste et al., 2009; Ratelle, et al. 2007), three or four sub-groups of adaptive to maladaptive profiles were expected. We also expected, consistent with Lens and colleagues’ instrumental goal research programme (i.e., Lens, Simons, & Vansteenkiste, 2004) to find goal temporal distance (distal compared to proximal) to be linked to adaptive profiles, especially when distal goals are internally regulated and hence reflect the personal importance of the goal. Specifically, we expected students with a profile high on internal goals to report the lowest levels of surface learning, highest levels of deep learning, and highest levels of GPA compared to other students.

As a final contribution, we aimed to test the person-centred implications of the Lens and colleagues’ instrumental goal framework (Simons et al., 2004) and complement previous Western research with a study in the context of a Japanese university. As such, we aimed to both expand the cultural borders of the field and also contribute to our understanding of an under-researched context. The Japanese model of compulsory and post-compulsory education is in broad alignment with the structure of Western education. Japanese university is four years in length and offers degree programs which most Western students and educators would recognise. The strongest contrast between the Japanese and Western model is the extremely high stakes entrance test exam Japanese students must take to enter specific faculties and the generally very high graduation rate (> 90%) from higher education. With regard to teaching and learning, teaching quality is consistent with Western universities; however, students might take between 8 and 15 different courses simultaneously (often each once a week for 90 minutes) leading to substantial curriculum crowding in some contexts. It is not clear what, if any, role the structure of curricula within Japanese higher education might play within students’ instrumental goal pursuit; it seems possible however, that it might enhance students’ focus on the proximal relative to distal reasons for studying at university.

## Methods

### Sample and Context

A total of 781 second-year students of different faculties from a medium sized private university in Western Japan participated in this study. All participants were aged 19 or 20. The sample included 256 female and 525 males, and was representative of the gender balance across the university’s population as a whole. Students completed the inventories during regular class time in about 15 to 20 minutes. Students participated after reading a description of the aims and nature of the broader study and were assured of their anonymity.

### Measures

All measures had been adapted and piloted in the Japanese university context (Fryer, 2013). Likert scale for survey items ranged from “Not at all like me” (1) to “Just like me” (6).

#### Instrumental goal measures

The instrumental goals were measured using a survey developed based on Lens and colleagues’ 2 × 2 framework (Simons et al., 2004), using discrete goals—relative to past continuum based measurement of these goals. This instrument has been developed across two different studies (e.g., Fryer, 2013; Fryer, et. al., 2013) and has been used successfully in two more recent variable-centred studies (e.g., Fryer, 2015, Fryer et al., 2014). We included the three goal types successfully used in these past studies in conjunction with social goals. Specifically, we asked students to think about their studying for their departmental courses when answering four items for proximal externally regulated goals (e.g., “I study to get my necessary credits; so that I can graduate”); four distal externally regulated goals items (e.g., “I study to make a higher salary in my future professional life”; “to become affluent and secure in my future adult life”); four distal internally regulated goal items (e.g., “so that in the future, I can use the skills I acquire in other domains than my job”; “to have a broader, clearer view of the world in my future”); and four socially instrumental goal items (e.g., “It gives me high esteem from my classmates”; “my parents and teachers tell me that my university studies are important”).

#### Amotivation

Student’s amotivation for studying was measured employing the Academic Amotivation Inventory (Legualt, et al., 2006). Three dimensions of amotivation (four items each) were selected for inclusion in the current study: task valuation (e.g., “I don’t study because my studies are not important to me”) ability beliefs (e.g., “I don’t study because the tasks demanded of me surpass my abilities”) and effort beliefs (e.g., “I don’t study because I can’t seem to invest the effort that is required”). This selection was based on previous variable-centred research in the context of departmental learning generally (Fryer, 2013) and research in specific environments such as e-learning (Fryer & Bovee, 2016; Fryer, Bovee, & Nakao, 2014). Given their high intercorrelations and the desire to measure amotivation generally these three variables were combined in the current study as a construct to represent general amotivation for studying (Legualt, et al., 2006).

#### Student outcomes

Students’ outcomes were measured in terms of approaches to learning and GPA. Approaches to learning were measured using deep approaches to learning (five items; e.g., “When I am working on a new topic, I try to see how all the ideas fit together”) and surface approaches to learning (five items; e.g., “I concentrate on learning just those bits of information I have to know to pass”). Approaches to learning scales were adapted and piloted across a series of studies (i.e., Fryer, 2013; Fryer, Ginns, Walker & Nakao, 2012) from a version of the Approaches to Study Inventory (Tait, Entwistle, & McCune, 1998) previously employed by Trigwell and Ashwin (2006). Finally, Grade Point Average for students’ second year studies was also included. This score was obtained for students who participated in the study from the university’s administration and was measured on a scale from 0 to 4.33.

### Analyses

Missing data (> 1%; Little’s MCAR test Chi-Square = 100.285, DF = 80, *p* = .062) was imputed employing LISREL 8.80 (Jöreskog & Sörbom, 2006). LISREL employs the EM algorithm, generating random draws from the probability distribution via Markov chains (for greater detail see Schafer, 1997). All latent analyses were conducted with M*plus* 7.0 (Muthén & Muthén, 1998–2014), employing its maximum likelihood robust algorithm. All other analyses were undertaken with JMP 9.01 (SAS, 2007–2011). Prior to conducting analyses, the imputed dataset was examined for extreme outliers (> 3 *SD*; Hadi, 1992).

As a starting point, a confirmatory factor analysis was conducted to ensure convergent and divergent construct validity. Fit for the model was based on one incremental (Comparative Fit Index; CFI) and one absolute (Root Mean Square Error of Approximation; RMSEA, including its 90% confidence interval) measure of fit. Acceptable/good fit was indexed with CFI values above .90/.95 (McDonald & Marsh, 1990) and RSMEA values below .05 (and below .08 for the upper bound of the 90% confidence interval) (Browne & Cudeck, 1992).

Latent Profile Analysis was employed for the study’s person-centered analyses using the four goal types discussed to this point and amotivation. Latent Profile Analysis (LPA) is a form of latent variable mixture modelling (Hagenaars, & McCutcheon, 2002). In contrast to the exploratory nature of cluster analysis (e.g., using Wards method; Vansteenkiste, Sierens, Soenens, Luyckx, & Lens, 2009), LPA is a confirmatory technique which a model-based solution provides a wide array of fit indices, while it generates probabilities for group membership (for a more complete discussion of LPA and standard clustering techniques within an educational context see Pastor, Barron, Miller, & Davis., 2006).

Fit for the latent profile analyses were assessed through the use of five fit indexes: two likelihood ratio tests and three information criterion indexes. With respect to the likelihood ratio tests, the Vuong-Lo-Mendell-Rubin Likelihood Ratio Test (Vuong, 1989) and Lo-Mendell-Rubin Likelihood Ratio Test (Lo, Mendell, & Rubin, 2001) both provide a test of whether the identified set of latent groups is less statistically significant than a solution with one group less, that is, whether the solution with one group less better fitted the data. The three information criterion indexes, Akaikes’s Information Criterion (AIC; Akaike, 1987), the Bayesian Information Criterion (BIC; Schwartz, 1978) and the sample size-adjusted BIC model represent each a selection criterion, wherein lower values indicate the preferred model. While all three information criteria have their weaknesses, the BIC is generally seen as being the most useful information criterion guide for LCAs (Nylund, Asparoutiov, & Muthen, 2007). In addition to these indexes, decisions regarding the optimal number of classes are also guided by the relative size of the classes, variance explained by the model and its theoretical meaningfulness.

Following latent profile analyses, a MANOVA was undertaken to assess the difference across finalized profiles. Follow-up ANOVAs were then conducted to examine differences between profiled variables across profiles. For these analyses *R*^2^ was reported to provide a measure of variance explained.

## Results

No extreme outliers were observed (> 3 *SD*; Hadi, 1992) and Skewness as well as Kurtosis were satisfactory for all variables. Based on the fit criteria described, a confirmatory factor analysis of all motivational and outcome variables together was judged to fit the data acceptably: χ^2^ = 1031 (*df* = 347), RMSEA = .05 (90% C.I. .047 – .054), CFI = .91. Table 1 provides the reliability for these scales (Cronbach’s alpha). These were acceptable for each of the measures, with the exception of surface approaches to learning (> .70; Devellis, 2012). Surface approaches to learning have an established history of low reliability (for a thorough review of this issue see Richardson, 1994), which is consistent with the current results. The inter-correlations of all observed variables are also presented in Table 1.

**Table 1:** Correlations and descriptive statistics for all variables modelled.

		1	2	3	4	5	6	7	8

1	Proximal externally regulated								
2	Distal externally regulated	.46**							
3	Distal internally regulated	.20**	.67**						
4	Socially regulated	.11*	.54**	.46**					
5	Amotivation	.11**	–.16**	–.43**	–.06				
6	Deep Approaches	–.08	.28**	.60**	.30**	–.27**			
7	Surface Approaches	.19**	.01	.19**	.06	.33**	.32**		
8	GPA	–.07	.07	.11**	.02	–.27**	.05	–.14**	
9	Gender (female = 1, Male = 2)	–.00	–.00	–.13**	–.02	.14**	–.11**	.00	–.30**

	MEAN	4.31	4.02	4.09	2.85	2.7	3.52	3.88	2.21
	SD	.83	.94	.85	.94	.82	.67	.65	.84
	Cronbach’s Alpha	.74	.78	.78	.78	.86	.74	.60	/
	Items	4	4	3	4	12	5	5	1

*Note:* * *p* < .05; ** *p* < .01. Measures 1–9 are measured on a scale of 1–6. GPA is measured on scale 0–4.33. Gender is female = 1 and Male = 2.

### Latent Profile Analysis

Fit for Latent Profile Analyses of two, three, four and five groups are presented in Table 2. Consistent with past research (Nylund, Asparoutiov, & Muthen, 2007) BIC provided the best guidance regarding optimal latent classes. While the Likelihood Ratio provided scant direction regarding the optimal number of classes, BIC, supplemented by reasonable group size (> 5%) and variance explained (> 50%), indicated that a three-class model fit the data best.

**Table 2:** Fit statistics for four Latent Profile Analysis conducted.

	Two Classes	Three Classes	Four Classes	Five Classes

Akaike Information Criterion (AIC)	9739.522	9573.673	9460.874	9376.572
Bayesian Information Criterion (BIC)	9814.152	9676.29	9591.477	9535.162
BIC Sample-Size Adjusted	9763.344	9606.429	9502.563	9427.195
Vuong-Lo-Mendell-Rubin Likelihood Ratio Test	0.765	0.825	0.88	0.805
Lo-Mendell-Rubin Likelihood Ratio Test	0	0.1	0.1829	0.523

Next, the explanatory power of this model was tested. A MANOVA with the three finalized classes as the independent and the profiled variables as the dependent variables was statistically significant: Wilks’ Lambda = .47 (*F* = 86.47, *p* < .0001. Follow-up ANOVAs tested for group differences the goals and amotivation. The mean levels for the three subgroups are presented in Table 3. There were statistically significant differences in amotivation between the first and second cluster on the one hand and the third cluster on the other hand; also, the three profiles differed to each other in all the four instrumental goals. The profiles explained 6% of the variance in amotivation and between 11 and 38% of the variance in the different goals. The three profiles could thus clearly be distinguished.

**Table 3:** Three Class Finalized Profile and Class-based ANOVA Results.

	Low future oriented (*n* = 51, 6.5%)	Average (*n* = 596, 76.0%)	Highly motivated (*n* = 137, 17.5%)	*p*	*F*	*R^2^*

**Goals**						
Distal internally regulated goals	3.20_a_(1.29)	3.92_b_(.62)	5.16_c_(.54)	< .0001	238.77	.38
Proximal externally regulated goals	4.00_a_(1.32)	4.20_b_(.73)	4.89_c_(.75)	< .0001	47.60	.11
Distal externally regulated goals	2.09_a_(.56)	3.88_b_(.59)	5.30_c_(.50)	< .0001	644.96	.62
Socially regulated goals	1.53_a_(.55)	2.78_b_(.78)	3.63_c_(1.04)	< .0001	131.76	.25
Amotivation	2.76_a_(.91)	2.80_a_(.77)	2.28_b_(.88)	< .0001	23.38	.06
**Outcomes**						
Surface approaches	3.87_ac_(.73)	3.79_a_(.61)	3.95_bc_(.77)	< .05	3.38	.01
Deep approaches	3.13_a_(.98)	3.39_b_(.57)	3.80_c_(.74)	< .0001	29.99	.07
GPA	2.05_a_(.86)	2.20_a_(.85)	2.33_a_(.82)	= .11	2.21	.01

Class N size	51	596	137			
Sample proportion	6.51%	76.02%	17.47%			
Female/Male class N size	16/35	181/415	59/78			
Female/Male N class proportion	32%/68%	30%/70%	43%/57%			

*Note:* Within row means with different letters are significantly (*p* < .05) different from each other.

The first profile included students who reported especially low scores on each of the different types of goals and relatively high scores on amotivation. In comparison to students in the second profile, students with this profile were particularly characterized by low distal goals (both internal and external) and socially instrumental goals. Compared to their counterparts in the first profile, students in the second profile reported higher scores for each of the goals, but equally high scores on amotivation. The third profile consisted of students characterized by lower scores on amotivation and high scores on each of the goals. Because of these results, we labelled the profiles subsequently, the low future oriented motivation profile, the average motivation profile, and the highly motivated profile.

**Figure 1:**
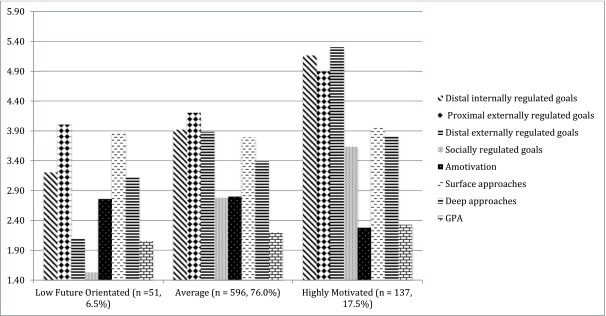
Profiled variables and covariates. *Note:* All variables presented, except GPA, are measured on a scale of one to six. GPA is measured on a scale of 0 to 4.33.

Following these analyses, difference testing was undertaken for outcomes. These are presented in Table 3. In Figure 1, the mean scores for each latent subgroup are presented for ease of visual comparison. The profiles explained differences in surface and deep level learning, but not in GPA. The only statistically significant difference across the groups concerning surface strategies was between the average motivation profile and high motivation profile. Although statistically significant, the profiles explained only 1% of the variance in surface learning. The profiles, however, clearly differed in deep learning. While students with the low future oriented profile reported the lowest levels of deep learning, students in the average motivation profile reported more deep level learning, while the students in the highly motivated profile reported the highest levels. Although the differences in GPA were consistent with the results for deep learning, no statistically significant differences (*p* < .05) were observed.

A final analysis examined gender differences across sub-group membership. The descriptive results are presented in Table 3. A chi-square test revealed a statistically significant, but small, difference in gender across the three profiles, with females being overrepresented in the high motivation profile (χ^2^ = 7.93, *df* = 2, *p* < .05, R^2^ = .01). The three latent groups each exhibited a reasonable proportion of the sample examined, with the highly motivated profile representing 17.5% of the sample, the average motivation profile representing 76.0%, and the low future oriented profile representing 6.5% of the sample.

## Discussion

The present study aimed to discover latent profiles of students based on their level of proximally versus distally and internally versus externally regulated goals and amotivation. Our results indicated that three groups best fitted the data. The three groups represented a low future oriented motivation profile with low distal goals and relatively high proximal goals as well as amotivation; an average motivation group, with middling amount of distal goals, and relatively high proximal goals and amotivation (much like the low future oriented group); finally, a highly motivated profile with the highest level of goals and the lowest amotivation. The largest differences were observed in students’ distal goals (both internal and external), where groups differed to each other in terms of deep level learning. In contrast, differences in surface learning were smaller and lacked a clear pattern, whereas no statistically significant differences were found for GPA across the three groups. Female students were over-represented within the high motivation profile and under-represented within the average profile.

The current results have implications for the literature on motivation. Researchers examining motivation from a person-centred perspective continue to debate the optimal number of subgroups in a population. The current study supports the proposition that while four groups may be theoretically possible according to Lens and colleagues’ 2 × 2 framework (Simons, et al., 2004), only a three-group solution was empirically identified as the current study failed to reveal a clearly low quantity profile. This is inconsistent with for example the results of Vansteenkiste et al. (2009) who studied clusters based on SDT’s autonomous and controlled motivation among high school and college students, but is consistent with Corpus and Wormington’s (2014) motivational profiles based on intrinsic and extrinsic motivation in elementary school children.

The fact that a subgroup with low motivation may not always be found is interesting and merits further examination. Our failure to find four groups may be due to several reasons. For example, it might have been due to the nature of the goals employed. In the current study, the goals sought to measure reasons for studying, reasons that students could relate to: graduation-oriented, job-oriented, future self-development-oriented and socially oriented. Particularly in the context of higher education, it may be quite unlikely that students would be low on all four goal pursuits. This might be particularly true in case of universities in Japan. While very important for students’ futures, higher education in Japan is non-compulsory; so students choose to come to university for some important reasons. This suggests that university students are at the very least motivated to complete university; an assumption that is supported by the relatively high levels of proximal externally regulated goals (compared to the other goals) in our low future oriented profile. The strong motivation to complete university in Japan is clearly supported by the national average graduation rates, which are consistently above 90% (OECD, 2014).

While the difference between the average and highly motivated group is mainly the difference in overall motivation reported, we do see the highly motivated students chiefly preferred distal over proximal goals. This seems to suggest that students who were strongly future-orientated were also more motivated. Notably, the students in the latter profile reported pursuing high levels of multiple instrumental goals for studying at university. Variable-centred research generally overlooks this reality.

The lack of significant difference in GPA observed across this study’s subgroups suggests that the employed measures of motivation herein are only weakly related to student performance. One potential reason for this lack of, what seems to be, a logical connection is the relative weak emphasis on achievement across a broad range of tertiary institutions in Japan. An example of this weak connection is the fact that nearly all students graduating from university in Japan find permanent employment well before the end of their fourth, and many before the end of their third year of study. It is unusual, therefore, for prospective employers to want to see transcripts of course grades during the hiring process.

A final result worth mentioning is that this study supports links between gender and motivation that have featured within the motivation literature in general (Voyer & Voyer, 2014), and specifically within the literature on autonomous/controlled goals (e.g., Ratelle et al., 2007). It also provides a person-centred perspective on past variable-centred research on gender and motivation employing these specific goal types (Fryer et al., 2014; Fryer, 2015). Female students were significantly more likely to pursue a profile of adaptive motivation and experience lower levels of amotivation. Despite only making up 33% of the entire sample, female students accounted for 43% of the high quality subgroup. While encouraging for female students, this result opens the question as to why male students were underrepresented in this group and how their motivation might be increased.

### Implications for practice

As reviewed, there is substantial research evidence suggesting that the type of motivation students display plays a substantial role in their persistence, learning and achievement (e.g., Simons et al. 2004). Against this background, the relatively small proportion of students who preferred distal goals (i.e., the highly motivated profile) is a cause for concern. Across a wide variety of faculties, most students from the current study (i.e., the average motivated profile) focused on the immediate curricular demands necessary for graduation. Two questions arise from this finding. First, what can educators and curriculum developers do to support future oriented motivation? Experimental research (e.g., Vansteenkiste, et al., 2004) suggests that even simple frames for tasks can have a substantial effect on students’ goals. In addition, lagged correlational modeling has indicated that prior self-concept can also play a substantial role in high and low quality goal pursuit (Fryer, 2015).

The second question—at the more structural level—is whether Japanese higher education is currently supporting students in making clear connections between learning and life beyond university. Substantial scholarship in the field of higher education (e.g. Amano & Poole, 2005; Takeuchi, 1997; Teichler, 1997) suggests that the connection between learning in higher education and future success may be relatively weak in Japan. It is therefore easy to understand why many students might not prefer distal goals, as they can expect in many cases to be entirely trained on the job within many Japanese corporations. Major Japanese corporations are well known for selecting new candidates based mainly on the reputation of the applicant’s university. As a partial result of these circumstances, there is the potential that students in such a context shift their focus to their immediate curricular demands as a means to graduate, which, as this study suggests, is related to a low quality learner profile.

By carrying out the present study at a Japanese university, we aimed both to begin to expand the cultural borders of the field and also contribute to our understanding of a specific under-research context (Japanese higher education). Findings from the current study indicate that person-centred goal research may be a useful approach for exploring Japanese students’ motivation as it is elsewhere in the world. Further research in this area might support reforms of key aspects of Japanese higher education, which the country continues to struggle with (see Amano, 2014; Yamada, 2014).

## Limitations and Future Directions

Care must be taken when drawing implications based on results from chiefly self-reported data. Evidence from this study is useful insofar as it fits into the fabric of the field of proximal and distal, externally and internally regulated goals. Due to the cross-sectional nature of the data, however, causal implication should not be drawn from the current findings.

The current study was undertaken at one mid-sized private Japanese university, and therefore prior to confidently generalizing to other institutions internationally or nationally, replication studies are required.

We thus call for more research, nationally and internationally, into the quality of motivations students come to university with and how it develops during students’ academic careers. Consistent with this call, it is important to note that as an applied field we have a responsibility to also assess practical constructs with clear relevance to the research context. The current study aimed to examine goals that were consistent with students’ reasons for studying in the researched context. Through careful development and piloting, measures can be constructed that are both context-sensitive and fit within the broader framework of goal regulation. We encourage future studies in this area to take a similar course of research so as to enlarge our theoretical and practical understanding of the various goals students may have for studying.

Future studies might include a measure of Future Time Perspective or Orientation. This would establish with greater clarity its potential role within the pursuit of multiple goals. Particularly in the case of future longitudinal studies, the role of FTP in goal development would be of interest. This approach might also disentangle questions regarding the benefit of multiple goal pursuit, which previous experimental research has suggested is not adaptive (Vansteenkiste et al., 2004), but is clearly common within student learning.

## Conclusions

Three goal profiles were observed in the current study. The highly motivated (high goal pursuit and low amotivation) students exhibited the most adaptive profile in terms of deep learning, compared to students being characterized by a low future oriented profile or an average motivated profile. We suggest, consistent with much of Willy Lens’ scholarship in this area, that education is by its nature future oriented, and that this fact, if properly integrated into the students’ internally regulated reasons for learning, can be a considerable source of adaptive motivation.
